# Practice variation in surgical procedures and IUD-insertions among general practitioners in Norway – a longitudinal study

**DOI:** 10.1186/s12875-017-0581-9

**Published:** 2017-01-21

**Authors:** Andreas Saxlund Pahle, Daniel Sørli, Ivar Sønbø Kristiansen, Trygve S. Deraas, Peder A. Halvorsen

**Affiliations:** 1 Bolteløkka legesenter, Sofiesgt 60, 0160 Oslo, Norway; 2 General Practice Research Unit, Department of Community Medicine, UiT- the Arctic University of Norway, P.O.Box 6050, Langnes, 9037 Tromsø, Norway; 3 Bankgården Legekontor, Sørumsandvegen 69, 1920 Sørum, Norway; 4 Department of Health Management and Health Economics, University of Oslo, P.O. Box 1089, Blindern, NO-0317 Oslo, Norway; 5 Center of Clinical Documentation and Evaluation, Northern Norway Regional Health Authority, Box 6, N-9038 Tromsø, Norway

**Keywords:** General practitioner, Primary health care, Surgical procedures, IUD-insertions, Practice variation, Comprehensiveness

## Abstract

**Background:**

Studies of Primary Health Care (PHC) reveal considerable practice variations in terms of the range of services provided. In Norway, general practitioners (GPs) are traditionally expected to perform IUD-insertions and several surgical procedures as a part of comprehensive PHC. We aimed to investigate variation in the provision of surgical procedures and IUD-insertions across GPs and over time and explore determinants of such variation.

**Methods:**

Retrospective registry study of Norwegian GPs. From a comprehensive database of GPs’ reimbursement claims, we obtained procedure codes and GP characteristics such as age, gender, list size and municipality characteristics from 2006 through 2013. Multivariable logistic regression models were fitted to explore determinants of practice variation.

**Results:**

We extracted data from 4,828 GPs. In 2013, 91.0, 76.1 and 74.8% were reimbursed at least once for minor and major surgical procedures and IUD-insertion, respectively. Female GPs had lower odds for performing major surgical procedures (OR 0.38, 95% CI 0.32–0.45) and higher odds for performing IUD-insertions (OR 6.28, 95% CI 4.47–8.82) than male GPs. Older GPs and GPs with shorter patient lists were less likely to perform surgical procedures. GPs with longer patient lists had higher odds for performing IUD-insertions. The proportion of GPs performing surgical procedures increased over time, while the proportion decreased for IUD-insertions. The number of IUD-insertions in specialist care increased from 12,575 in 2011 to 15 216 (+21.0%) in 2014.

**Conclusion:**

We observed a large variation in the provision of surgical procedures and IUD-insertions amongst GPs in Norway. The GPs’ age, gender, list size and size of municipality were associated with performing the procedures. Our findings suggest a shift of IUD-insertions from primary to specialist care.

## Background

For many decades, health care researchers have shown large variations in the utilization of various health services within countries and regions. As some of these variations are sizeable, concerns have been raised regarding the quality and equity in the delivery of health care [1–3]. Examples of practice variation in Primary Health Care (PHC) include utilization of spirometry in COPD-patients and prescriptions of antibiotics in patients with sore throats [4–8]. Wide variations in referral rates are also seen in countries with a GP gatekeeping function. This variation is largely unexplained although some of it may reflect patient and GPs preferences [8–11]. One of the proposed frameworks for practice variation research is the “supply hypothesis” in which the doctor’s role as an agent acting on the patient’s behalf influences medical decision making [12]. Two analytical traditions exist under this hypothesis: 1) an economic model where decisions are motivated by income (the supplier-induced demand-theory) and 2) a model were doctors develop different practice styles to cope with the uncertainty inherent in clinical practice.

The studies of practice variations pertain to one of the traditional core virtues in PHC - its comprehensiveness. While the traditional scope of general practice has been to take care of almost all of the patient’s health care needs, evidence shows sizable variations and also reductions in what services are offered [6, 13]. In 1996, the Institute of Medicine defined comprehensiveness as “*the provision of integrated, accessible health care services by clinicians who are accountable for addressing a large majority of personal health care needs”* [14] and in 2008 the World Health Organization supported its value [15]. Even though a comprehensive service might be an ideal, studies from countries comparable to Norway show an increasing fragmentation and declining comprehensiveness in PHC. In Ontario, GPs make fewer house calls and work more exclusively in their surgeries [16]. In British Colombia, comprehensive geriatric and obstetric care by GP declined over a 20-year study period [17]. In Denmark and Norway, there has been an increase in referrals of patients to secondary care over the last two decades, a phenomenon that might reflect a similar decline in comprehensive GP care [10, 18].

### Health Care in Norway

In 2001, the Norwegian government introduced a PHC-list patient system that covers almost all GPs and 99.2% of the population [19]. The GPs are reimbursed by a combination of capitation (approximately 30% of GPs’ gross income), service fees (approximately 40%) and patient co-payments. Almost all GPs in Norway are a part of this reimbursement system; only 5.9% receive a salary [19]. Approximately half of all GPs in Norway are certified specialists in general practice [20]. The Norwegian GPs function as gatekeepers between primary – and specialist health care in order to ensure equity and efficiency through “fair rationing” [21]. GPs receive fees for a range of services including various types of surgical and gynaecological procedures. Many reimbursable services, such as IUD insertion, have a unique reimbursement code [22]. Other codes encompass more than one service or procedure (Table 1). All codes are registered in a dedicated database at the Norwegian Health Economic Administration (HELFO), which registers GPs invoices electronically on a bimonthly basis.

**Table 1:** Overview of reimbursement codes 100 and 105

Code 100, some examples:	
-Treatment of epistaxis	
-Skin biopsy	
-Removal of foreign body from eye and ear/nose/thro	
-Implantation of medical implants	
-Injection of medication in joints and tendon sheath	
-Surgical removal of small skin tumours	
-Wound treatment with/without sutures	
-Arterial bloodgas sampling	
-Cleaning of external Auditory Canal (IE Cerumen Removal)	
Code 105, some examples:	
-Incision of abscess	
-Urinary catheterization and bladder washout	
-Surgical removal of small subcutaneous tumours, nail, large nevus or nevus from face	
-Ligature of haemorrhoid	
-Puncture of joint and pleura for sampling	
-Lumbar puncture	
-Larger wound treatment and debridement	
-Advanced treatment of chronic wounds	

Specialist Health Care (SHC) in Norway is mainly offered within a public hospital setting, but also by private specialists who are reimbursed publicly.

### Study aim

The aim of this study was to investigate practice variations amongst Norwegian GPs by analysing the number of surgical and gynaecological procedures per year per GP. Our thought was that by identifying quantifiable indicators of what we consider to be among Norwegian GPs core activities; surgical procedures (as presented in Table 1) and IUD-insertions, we aimed to: 1) Explore practice variations amongst Norwegian general practitioners when performing the procedures and 2) explore change in these procedures over a time period. We also wanted to explore whether there has been a shift of IUD-insertions from PHC to SHC.

## Methods

### Subjects

From the HELFO database we identified all GPs working in daytime surgeries from 2006 through 2013 (*n* = 5218). Because substitute GPs (for instance seasonal workers) might have a different work profile, we excluded GPs with < 1000 consultations annually (approximately 7.5% of the cohort). In total, we included 4828 GPs in our study sample. To ensure anonymity of the physicians, HELFO gave each GP an anonymous ID.

### Data collection

From the HELFO database we collected information on GP characteristics (age, gender, list size and municipality). Also, for each GP, the following procedure codes, as listed in the “Fee schedule for Norwegian physicians” (in Norwegian: Normaltariffen) [23], were extracted: code 100 (minor surgical procedures), code 105 (major surgical procedures) and code 214a (insertion/change of IUD and insertion of birth control implants). Table 1 shows a listing of some of the minor and major surgical procedures that are covered by procedure codes 100 and 105. Code 100 includes many procedures; of which some can be performed by trained nurses. Hence, to restrict code 100 to minor surgical procedures performed by GPs themselves, we only included claims in which code 100 was accompanied with 149a, the code for local anaesthesia, which is expected to be performed simultainously. All data were extracted using JasperSoft. Statistics Norway provided information on population size for each municipality as of January 1, 2014. The Norwegian Patient Register (NPR) holds information on all episodes of care in specialist health care including private specialists (in-patient, out-patient and day-care). From this register we obtained data on all IUD insertions (procedure code TLC00) for the years 2011–2014. From the Norwegian Prescription Register we extracted the number of hormone IUDs delivered from pharmacies for each year of the study period.

### Statistical analysis

Baseline characteristics of the participating GPs and primary outcome measures were described in terms of means and proportions. Our primary outcome measures were whether or not the GPs had claimed reimbursement for minor surgery (codes 100 + 149a), major surgery (code 105) or IUD-insertion (code 214a), respectively. Each GP was scored one if she or he had performed the procedure, otherwise 0. We developed logistic regression models to explore possible determinants of use of the respective codes. Independent variables were GP age, GP gender, list size and municipal population size. We tested for first order interactions that were considered plausible (age/list size, gender/list size and gender/age) by adding product terms to the regression models. To avoid potential within-subject variation by time we performed regression analysis for every procedure for each year of the study period. As the trends in the models were the same for all years, we only present the 2013 data*.* All statistical analyses were performed in SPSS, version 21 and Excel, version 14.2.1.

## Results

Baseline GP characteristics in 2006 and 2013 are shown in Table 2. In 2013, 91.0% of GPs performed at least one minor surgical procedure, 76.1% at least one major surgical procedure and 74.8% at least one IUD-insertion.

**Table 2:** Baseline characteristics for participating GPs in 2006 and 2013

	2006	2013
Number of participants	3015	3609
Female gender	28.6%	35.6%
Average list size	1254	1213
Age		
< 40	17.7%	21.8%
41–50	30.0%	25.5%
51–60	39.5%	30.5%
> 60	12.7%	22.1%
Proportion performing procedures		
Minor surgery, (overall mean)	87.5% (31)	91.0% (34)
Major surgery, (overall mean)	67.9% (13)	76.1% (16)
IUD-insertions, (overall mean)	80.4% (7)	74.8% (5)

### Minor surgical procedures (reimbursement codes 100 + 149a)

The crude number of minor surgical procedures was 93,430 (performed by *n* = 3015 GPs) in 2006 and 122,437 (*n* = 3609), in 2013 (+31.0%). The Norwegian population grew by 8.9% over the same period. The mean number of minor surgical procedures per GP per year was 31.0 in 2006 and 33.9 in 2013, (*+*9.7%). In 2006, 82.5% of female GPs and 89.5% of male GPs performed at least one minor surgical procedure. The corresponding numbers in 2013 were 88.6 and 92.3%. There was an increase in mean number of performed procedures for both genders from 2006 to 2013 (19.7 versus 25.0 for female GPs and 35.5 versus 38.9 for male GPs). In regression analysis of 2013 data, female GPs had lower odds of performing the procedures (OR 0.58 95% CI 0.45–0.74) compared to male GPs (Table 3). Older GPs had lower odds of performing minor surgical procedures (Table 3). There were no statistically significant interactions.

**Table 3:** Multivariable logistic regression models for Norwegian GPs claiming reimbursement for minor surgery, major surgery and IUD-insertions in 2013

	Minor surgery	Major surgery	IUD-insertion^a^
	(OR 95% CI)	(OR 95% CI)	(OR 95% CI)
GP gender			
Male (ref)	1	1	1
Female	0.58 (0.45–0.74)	0.38(0.32–0.45)	6.28 (4.47–8.82)
GP age			
< =40 (ref)	1	1	1
41–50	0.68 (0.46–1.02)	0.86(0.68–1.10)	1.17 (0.98–1.49)
51–60	0.55 (0.38–0.81)	0.68(0.54–0.87)	1.10 (0.88–1.38)
> 60	0.33 (0.22–0.48)	0.46(0.36–0.60)	0.92 (0.73–1.17)
GP list size			
< 1000 (ref)	1	1	1
1001–1200	0.97 (0.68–1.39)	1.21(0.97–1.52)	1.82 (1.42–2.39)
1201–1500	0.92 (0.66–1.28)	1.44(1.15–1.79)	1.78 (1.39–2.28)
> 1500	1.09 (0.73–1.62)	1.74(1.32–2.30)	2.46 (1.85–3.26)
GP municipal size			
< 5000 (ref)	1	1	1
5001–10,000	2.74 (1.50–5.01)	0.91(0.63–1.32)	1.41 (1.02–1.94)
10.001–25.000	1.88(1.16–3.03)	0.84(0.60–1.17)	1.65 (1.23–2.21)
25.001–100.000	1.24 (0.80–1.93)	0.55(0.40–0.76)	1.06 (0.80–1.41)
> 100.000	0.65 (0.42–1.00)	0.39(0.28–0.54)	1.02 (0.76–1.37)

^a^Adjusted for the interaction term gender x list size. This interaction is outlined in Fig. 1

### Major surgical procedures (reimbursement code 105)

The crude number of major surgical procedures was 39 034 (*n* = 3015) in 2006 and 57 065 (*n* = 3609), in 2013 (+46.2%). The mean number per GP per year was 13.0 in 2006 and 15.4 in 2013(+23.1%). In 2006, 50.5% of female GPs and 74.9% of male GPs performed at least one major surgical procedure. The corresponding numbers in 2013 were 64.9 and 82.3%. There was an increase in the mean number of procedures performed for both genders from 2006 to 2013 (5.3 versus 7.7 for female GPs and 16.0 versus 19.7 for male GPs). In regression analysis of 2013 data, female GPs (OR 0.38 95% CI 0.32–0.45), older GPs, GPs with short patient lists and GPs working in the largest municipalities had lower odds of performing major surgical procedures (Table 3). There were no statistically significant interactions.

### IUD-insertions (reimbursement code 214a)

The crude number of IUD-insertions was 20,188 (*n* = 3015) in 2006 and 19,393 (*n* = 3609), in 2013 (− 4.0%). The mean number per GP per year was 6.7 in 2006 and 5.4 in 2013 (−28.6%). In 2006, 94.0% of female GPs and 75.0% of male GPs performed at least one IUD-insertion. The corresponding numbers in 2013 were 89.3 and 66.8%. There was a decrease in the mean number of performed procedures for both genders from 2006 to 2013 (11.5 versus 9.0 for female GPs and 4.8. versus 3.4 for male GPs). In regression analysis of 2013 data, female GPs (OR 6.28 95% CI 4.47–8.82) and GPs with larger patient lists (1500 patients or more versus less than 1000 patients, OR 2.46 95% CI 1.85–3.26) had higher odds of performing the procedures (Table 3).

**Fig. 1:**
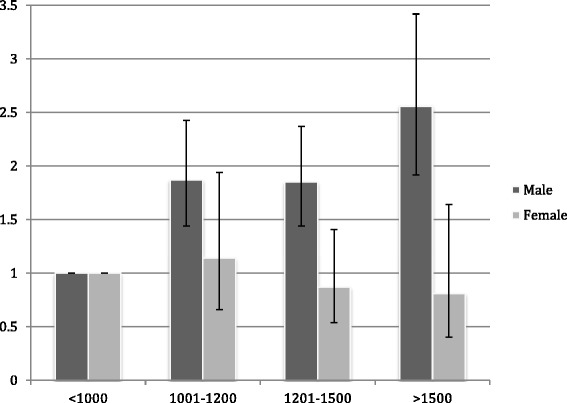
Difference in OR of performing IUD-insertion stratified by GPs sex and list-size (95% CI)

There was a statistically significant interaction between gender and list size. Stratification showed that the odds for performing the procedure increased with increasing list size among male GPs, while this effect was not present for female GPs (Fig. 1)

### Sensitivity-analysis

To explore whether our findings were sensitive to criteria for inclusion and exclusion of GPs into the study, we repeated all analyses in the entire cohort (*n* = 5218) and for GPs working all eight year of the study period (*n* = 2378). We found the same trends as in the main analyses (data not shown).

### Norwegian Patient Registry and Norwegian Prescription Registry

Data from the Norwegian Patient Registry indicate a continuous increase in the number of IUD-insertions in specialist care, from 12,575 in 2011 to 14,760 in 2013 (+17.4%) in women without concomitant abortion. For women with concomitant abortions there was a decrease over the same period from 808 to 729. Data from the Norwegian Prescription Registry show that the annual number of redeemed prescriptions for hormone IUDs was 9.83 per thousand women in 2006 and 10.1 per thousand women in 2013. The annual number of redeemed prescriptions for birth control implant was 0.66 per thousand women in 2006 and 2.63 per thousand women in 2013.

## Discussion

We observed sizable variations in terms of provision of surgical procedures and IUD-insertions among Norwegian GPs. While a majority of GPs did perform the procedures, many did not. Among those GPs who *did* perform the procedures, we found considerable variations in the number of procedures per year per GP. The GPs’ age, gender, list size and size of municipality were associated with performing the procedures. Our study also showed a relative increase in the practice of surgical procedures and a decrease in IUD-insertions during the study period. Over the same period, there was an increase in IUD-insertions in specialist care.

### Strengths and limitations

This study’s major strength is that it encompasses the entire GP population in Norway over an eight-year period. Another strength is the connection between complete data sets from Primary Health Care, Specialist Health Care and the Norwegian Prescription Registry, which expand the perspectives for a variation study.

One potential limitation lies in the assumption that the use of procedure codes reflects real practice among GPs. However, as 95% of the GPs in Norway work within a fee-for-service system, they have an incentive to include actual procedures in their reimbursement claims. Another potential limitation of this study may be the strict criteria for including GPs in the study. The criteria were chosen to examine “stable” GPs caring for patients they know. It has recently been shown that there is much turnover among GPs in Norway [24]. However, supplementary analyses of a wider selection of GPs did not change for the more restricted group of GPs. The third limitation is that the code 214a includes insertion/change of both IUD and birth control implants. It is not possible to distinguish between the two in this study, but as as noted above (page 11), the number of redeemed prescriptions for birth control implants increased from 2006 to 2013, but was still way below the number of prescriptions for hormone IUDs. However, the only way the increased prescription rate of birth control implants could influence our results is by confounding an even steeper decline in IUD-insertions throughout the period.

### Interpretation and comparison with existing literature

#### Practice variation

We are not aware of any studies concerning practice variation of surgical and gynaecological procedures by GPs inside PHC, but our findings of sizeable variations in services provided by health care providers is in line with other studies in this research field [1–3, 5, 7, 8, 25]. The main determinant of practice variation in our study was GP gender. In a recent study, GP gender was associated with referral rates to specialist health care (female GPs referred more often than male GPs) [10]. As we had no data on patient characteristics, we were unable to adjust for case-mix differences between female and male GPs, a mechanism proposed to explain some of the variation in previous studies [26, 27].

From a theoretical viewpoint the observed variations in surgical and gynaecological procedures by GP characteristics (age, gender, list size) could reflect difference in practice style (i.e. the clinical uncertainty theory [12]). Arguably, supplier-induced demand seems less likely since the procedures we studied would have very limited effect on the GPs’ gross income. For IUD insertions, patient preferences might explain why female GPs with more female patients performed more insertions [28]. The decreasing incidence of the IUD-procedures among GPs over the study period might be explained by an increasing supply of specialized health care especially in the larger cities. Consequently, supplier-induced demand from specialist could explain our finding. Regarding our findings that GPs working in the largest municipalities have lower odds of performing major surgical procedures, this seem to align well with a study from Iowa. Here, GPs working in nonmetropolitan settings performed more services including surgical procedures than GPs working in metropolitan areas [25]. Interestingly, Rivet et al. found that a larger range of procedures was associated with a higher level of job satisfaction among primary care physicians [29]. As Weigel et al. pointed out, this insight could perhaps inform strategies for recruitment of GPs to rural and remote areas [27]. Regarding the overall practice variation, many possible explanations must be considered, including changing patient preferences (i.e. patients prefer treatment and follow-up from specialists) or increasing fear of mistakes by GPs who seldom perform the procedures (i.e. risk aversion or defensive medicine) [30–33].

### Comprehensiveness

About one in four GPs did not perform surgical procedures or IUD-insertions. We do not know whether this indicates that GPs’ definition of comprehensive services is narrowing, patients increasingly prefer specialist care or simply if GPs restrict their portfolio of services for convenience or economic reasons. While a recent study from O’Malley described a declining comprehensiveness in the U.S. and Canada, there are also studies that suggest otherwise in the Netherlands [17, 34, 35]. Our findings could be interpreted as a sign of increasing fragmentation or declining comprehensiveness of PHC. Interestingly, Hobbs et al. argued that the clinical workload in UK PHC seems to have reached a threshold, indicating that PHC is in fact too comprehensive with too many tasks [36]. The increasing workload and task shift over the last decade, might therefore be forcing GPs to prioritize their work based on 1) their patients’ needs and 2) what the GPs deem more important in PHC.

Our findings suggest an increased referral rate of IUD-insertions to specialist care. This aligns well with other recently published studies. A study from Denmark, which has a well-organized primary care comparable to Norway, and a study from Norway showed that the overall rates of referral to secondary care have increased over the last two decades [10, 18].

### Implications for practice and research

The results of this study show that GPs in Norway did not offer the same surgical and gynecological procedures to their patients in the studied time period. Our findings raise several questions for practice and research: Should surgical and gynecological services be considered an integral part of comprehensive primary health care? If so, should all GPs be required to offer these services? Further research should focus on also other procedures and services in PHC, and qualitative studies among those who perform procedures and those who do not to shed some light on GPs’ priorities.

## Conclusion

We observed a large variation in the provision of surgical procedures and IUD-insertions amongst GPs in Norway. The GPs’ age, gender, list size and size of municipality were associated with performing the procedures. Our findings suggest a shift of IUD-insertions from primary to specialist care.

## Abbreviations

GP: General practitioner
HELFO: The Norwegian Health Economic Administration
IUD: Intrauterine device
NPR: The Norwegian Patient Register
PHC: Primary Health Care
REK Nord: Regional Committee for Medical and Health Research Ethics in Northern Norway
SHC: Specialist Health Care
